# Spontaneous regression of a primary squamous cell lung cancer following biopsy: a case report

**DOI:** 10.1186/s13256-018-1589-z

**Published:** 2018-03-12

**Authors:** Nathan Esplin, Khadija Fergiani, Timothy B. Legare, John W. Stelzer, Hammad Bhatti, Sayed K. Ali

**Affiliations:** 1 University of Central Florida College of Medicine, 6850 Lake Nona Boulevard, Orlando, FL 32827 USA; 2 Department of Internal Medicine, Orlando VA Medical Center, 13800 Veterans Way, Orlando, FL 32827 USA

**Keywords:** Spontaneous regression, Squamous cell carcinoma, Immune response

## Abstract

**Background:**

Spontaneous regression has been defined as occurring when the malignant tumor mass partially or completely disappears without any treatment or as a result of a therapy considered inadequate to influence systemic neoplastic disease. Recently, studies have implicated immunological responses as likely being involved. We report a case of a patient with squamous cell carcinoma of the lung who experienced spontaneous regression following biopsy without other intervention.

**Case presentation:**

A 57-year-old white man was referred to our pulmonary clinic after an incidental finding of a nodule in the lower lobe of his left lung. Thoracic computed tomography revealed a 2.0 × 1.4 × 1.5 cm spiculated nodule in the superior segment of the left lower lobe. Workup identified the mass as a squamous cell carcinoma that was clinically staged as T1M0N0. The patient deferred treatment of this lesion. He undertook no significant lifestyle or medical changes. Three months later, computed tomography revealed that, compared with the initial study, the solitary mass had decreased in size to 1.6 × 0.9 × 0.9 cm. Follow-up computed tomography 1 year after the original workup demonstrated that the nodule had stabilized to its smaller size.

**Conclusions:**

Studies have shown that immunological response can be initiated by trauma to an area. Because the tumor regression became evident in our patient only after the tissue biopsy, his immune response to the surgical procedure seems to be a plausible contributor to the spontaneous regression. Further understanding of spontaneous regression can potentially impact the identification of neoplastic drug targets or even the course of a patient’s treatment plan and goals.

## Background

Spontaneous regression (SR) of a tumor was originally defined as occurring “when the malignant tumor mass partially or completely disappears without any treatment or as a result of a therapy considered inadequate to influence systemic neoplastic disease” [1, 2]. SR of primary malignant lung tumors remains a rare occurrence [1, 3]. The precise mechanism behind SR is a focus of ongoing research. Recent studies have revealed a possible influence of various processes, including immune mediation, tumor inhibition by cytokines or growth factors, hormonal influence, elimination of carcinogenesis, tumor necrosis, angiogenesis inhibition, apoptosis, epigenetic mechanisms, and induction of differentiation [2–5]. We present a case of a patient with primary squamous cell lung cancer, stage T1M0N0, that demonstrated SR following biopsy with no additional therapeutic intervention by the medical team or lifestyle change by the patient.

## Case presentation

A 57-year-old white man was seen in the pulmonary clinic of our institution after an abdominal/pelvic computed tomographic (CT) scan for microscopic hematuria incidentally demonstrated a nodule in the left lower lobe (LLL) of his lung. The patient reported periodic chronic cough but denied any hemoptysis or any other alarming symptoms. His comorbidities consisted of hypertension, hyperlipidemia, latent syphilis, obesity, and diabetes mellitus type 2 with peripheral neuropathy. His daily medications included aspirin, lisinopril, hydrochlorothiazide, gemfibrozil, metformin, glipizide and amitriptyline. He reported a greater than 40-year history of smoking an average of two packs of cigarettes per day. He lived alone at home and was able to perform all of his activities of daily living. He reported that both his parents had died from lung cancer due to tobacco use. His physical examination and laboratory results had no pertinent abnormal findings.

**Fig. 1:**
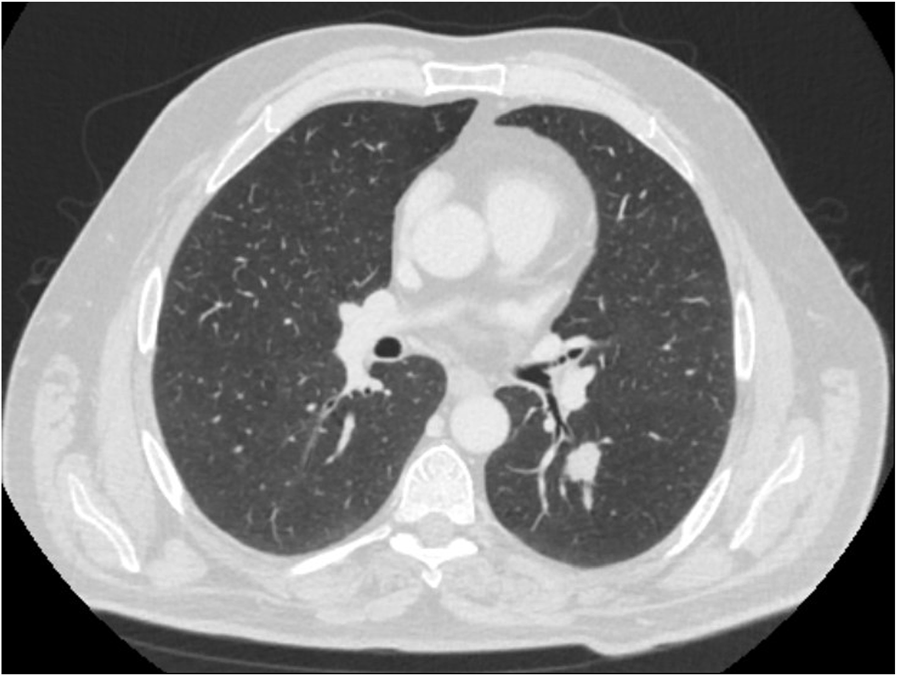
Thoracic computed tomographic scan taken in April 2016

A thoracic CT workup in April 2016 revealed a 2.0 × 1.4 × 1.5 cm spiculated nodule in the superior segment of the LLL (Fig. 1). This solitary nodule did not extend into the pleural surface, and no regional lymph node enlargement was noted. Bronchoscopy with alveolar lavage revealed malignant cells. On a positron emission tomographic (PET) scan, the pulmonary nodule had mild fluorodeoxyglucose (FDG) uptake that was indicative of metabolic activity resembling a low-grade neoplasm. The lesion was biopsied, and the result of immunohistochemical staining was negative for thyroid transcription factor-1 but positive for p63 and p40, correlating with squamous cell carcinoma. An area within the patient’s right suprapubic ramus also demonstrated abnormal FDG uptake on the PET scan, but subsequent bone marrow biopsy of that region revealed no evidence of metastatic cancer. On the basis of these findings, his lung carcinoma was clinically staged as T1M0N0.

**Fig. 2:**
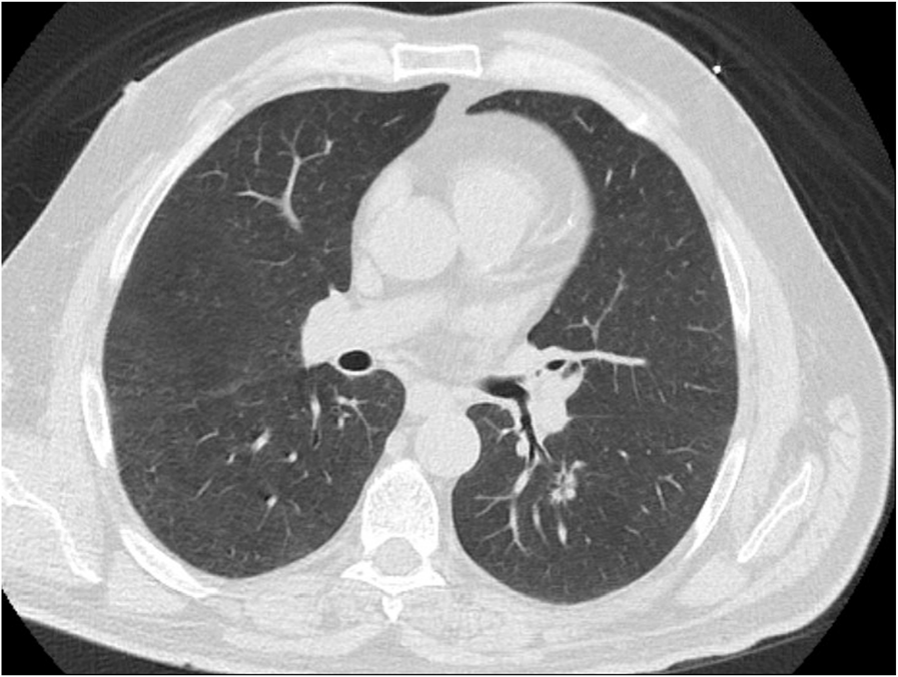
Repeat thoracic computed tomographic scan taken in July 2016

Surgical resection of the LLL was recommended on multiple occasions, but it was refused by the patient. He refused additional workup or treatment and reported no change in lifestyle. Three months later, in July 2016, a repeat CT scan revealed that, compared with its appearance on the initial chest CT scan, the solitary mass had decreased in size to 1.6 × 0.9 × 0.9 cm (Fig. 2). The patient underwent computed tomography a third time in August 2016, which revealed that the nodule had stabilized at its smaller size.

## Discussion

The widely accepted criteria for SR were published by Cole and Everson in 1956 [1]. They defined SR as “the partial or complete disappearance of a malignant tumor in the absence of all treatment, or in the presence of therapy which is considered inadequate to exert a significant influence on neoplastic disease” [1]. A literature search revealed the rare occurrence of SR in various cancer types, such as melanoma, renal cell carcinoma, and neuroblastoma [3]. There are only a handful of cases in the literature in which authors have reported SR in a case of primary lung cancer [1, 6, 7]. Lopez-Pastorini and colleagues [7] reported a case of presumed squamous cell cancer that demonstrated SR after biopsy of a mediastinal lymph node, but they were unable to acquire tissue from the lung mass itself. They also summarized older articles that show just how scarce is the number of reported primary lung cancer SR cases; one such article they referenced described only two reports of primary lung cancer SR between 1951 and 2008 [7].

Investigations on the mechanisms behind tumor SR have been inconclusive, but there are several theorized mechanisms. These studies and case reports have postulated different processes, such as immune mediation, tumor inhibition by cytokines or growth factors, hormonal influence, elimination of carcinogenesis, tumor necrosis, angiogenesis inhibition, apoptosis, epigenetic mechanisms, and induction of differentiation [2–4, 8]. A leading hypothesis is that immunological response can be initiated by trauma to the area, such as when a tissue biopsy is extracted [5]. In their original report, Cole and Everson showed that 40% of their cases of SR were related to some type pf operative trauma, and they concluded that stimulation of the immune process plays a key role in SR of cancers [1, 7]. Butterfield’s review on cancer vaccinations mentions how surgery and tumor-ablative procedures of various magnitudes can be considered a sort of “cancer vaccine” [9]. This comparison is attributed to how an ablation of a mass can affect antitumor immunity by the release of immunologically active tumor antigens that are inevitably liberated by damaged and dying cells during the ablation process [9]. Other authors report an “abscopal” effect in which radiation to one mass can lead to regression in a mass that was not in the field of radiation [10]. Mechanisms for this effect are thought to be mediated by the release of tumor antigens, “danger signals” (e.g., heat shock proteins and high morbidity group box 1), and proinflammatory cytokines [11]. This abscopal effect further implies that insult to cancer cells may initiate an immune response that acts more widely than the area of insult, and efforts are underway to further understand and potentially treat cancer with this mechanism by combining insult to cells and immunotherapy in the form of injecting “danger signal” proteins and proinflammatory cytokines [8, 11].

The lung cancer in our patient was essentially an incidental finding based on a CT scan, and the patient reported no significant symptoms. On the basis of current standards of care, surgery was recommended, but the patient declined. This aspect differs from other cases where patients had a lung cancer workup because of their complaints of progressive symptoms or because less sensitive imaging modalities (e.g., chest X-rays) had evident findings, indicating that the mass was potentially quite extensive/severe or metastatic. Our patient’s cancer was clinically stage I. Patients in other case reports had more advanced stages [6, 7]. Case reports involving different stages of a tumor help further characterize SR and how it can occur in any stage of cancer. In addition, continuing to report patients who experience SR under any circumstances can potentially impact the identification of neoplastic drug targets or other novel treatments of cancer. Our patient’s case reinforces the need to further understand the mechanisms of tumor regression and the potential for treatment modalities that may significantly reduce the morbidity and mortality of cancer.

## Conclusions

The case of our patient meets the SR criteria discussed in this report. The workup of the nodule revealed squamous cell carcinoma of the lung in a very early stage. Surgical resection is the standard of care and was recommended to the patient multiple times, but he declined. It is inadvisable to recommend watchful waiting in a case such as this one, but our patient’s refusal of surgery allowed for the opportunity to follow this tumor after biopsy. Repeat imaging 3 months after biopsy with no further treatment revealed shrinkage of the mass. During this period, the patient also denied any changes in his daily medications or lifestyle. Because the tumor regression became evident after the tissue was biopsied, the patient’s immune response to the surgical procedure seems to be a plausible factor in the occurrence of SR in his primary lung tumor. Our patient with a tumor that would typically be resected but was followed with imaging after biopsy demonstrates the potential for regression following insult or damage to a tumor, and this case report adds to the growing body of knowledge that implies the possibility of effective and potentially novel immunological treatments of cancer.
